# Supplementary material for: Glomerales Dominate Arbuscular Mycorrhizal Fungal Communities Associated with Spontaneous Plants in Phosphate-Rich Soils of Former Rock Phosphate Mining Sites
Source: Microorganisms. 2022 Dec 5;10(12):2406. doi: 10.3390/microorganisms10122406 (PMC9782746; doi:10.3390/microorganisms10122406)
Supplement: Supplementary file 1 [file microorganisms-10-02406-s001.zip › microorganisms-1977653-supplementary.pdf]

SUPPLEMENTARY DATA

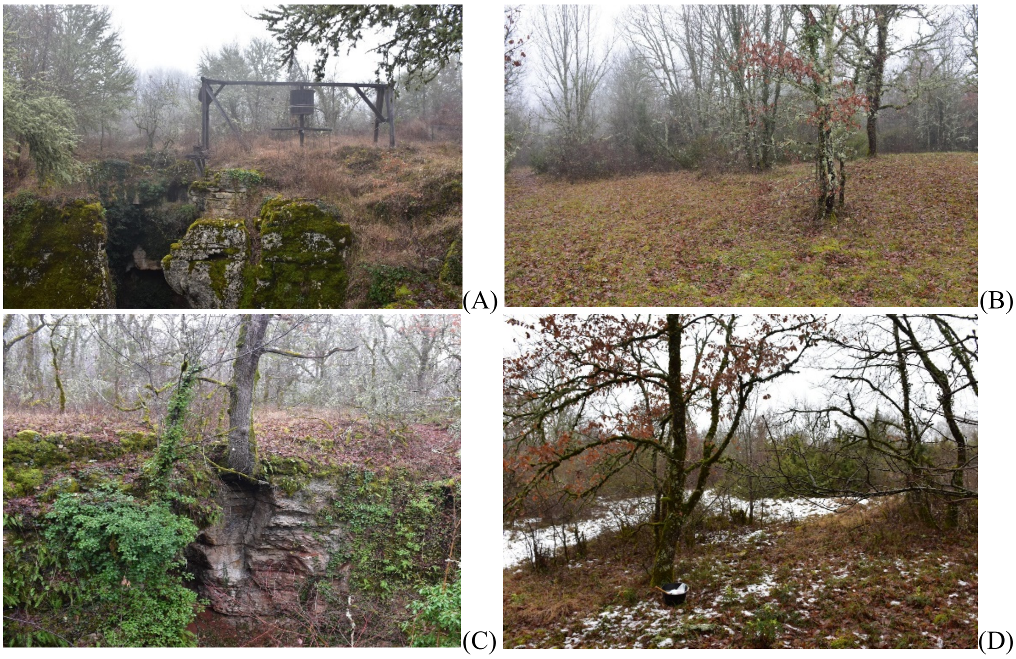

Figure S1: A: the remaining material from the past open-pit mines, B, C, D: Landscape in location 1, 2 and 3 respectively

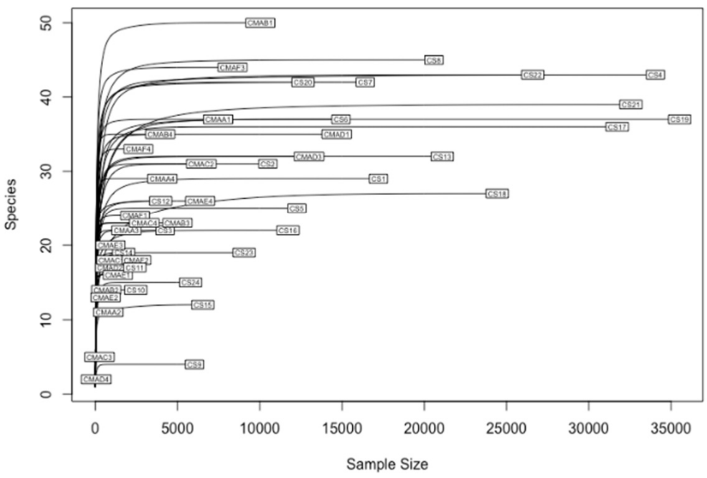

(A)

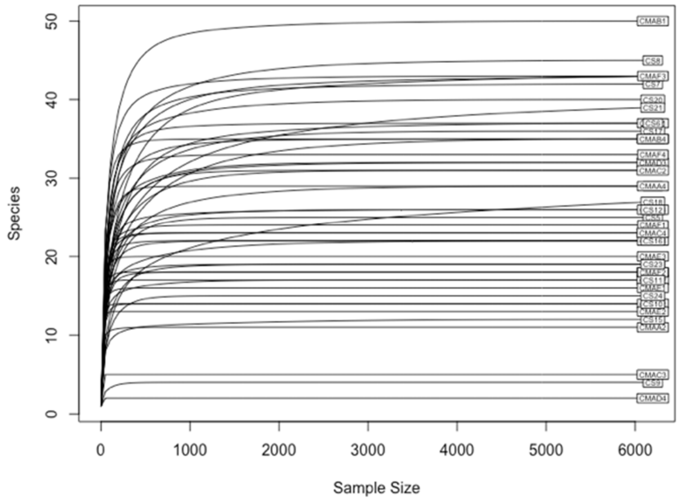

(B)

Figure S2: Rarefaction curves obtained from the 18S rRNA gene dataset.

(A) Curves obtained from the 3.527.278 sequences yielded after quality filtering and chimera removal (24 soil samples and 24 root samples). (B) Rarefaction curves obtained after a random resampling step, computed to compensate for the uneven sequencing efforts of different samples and to keep an adequate sequencing depth to capture most of the AMF diversity from soil and root samples. Thus, the curves were obtained for a standardized 6 000 sequences per sample.

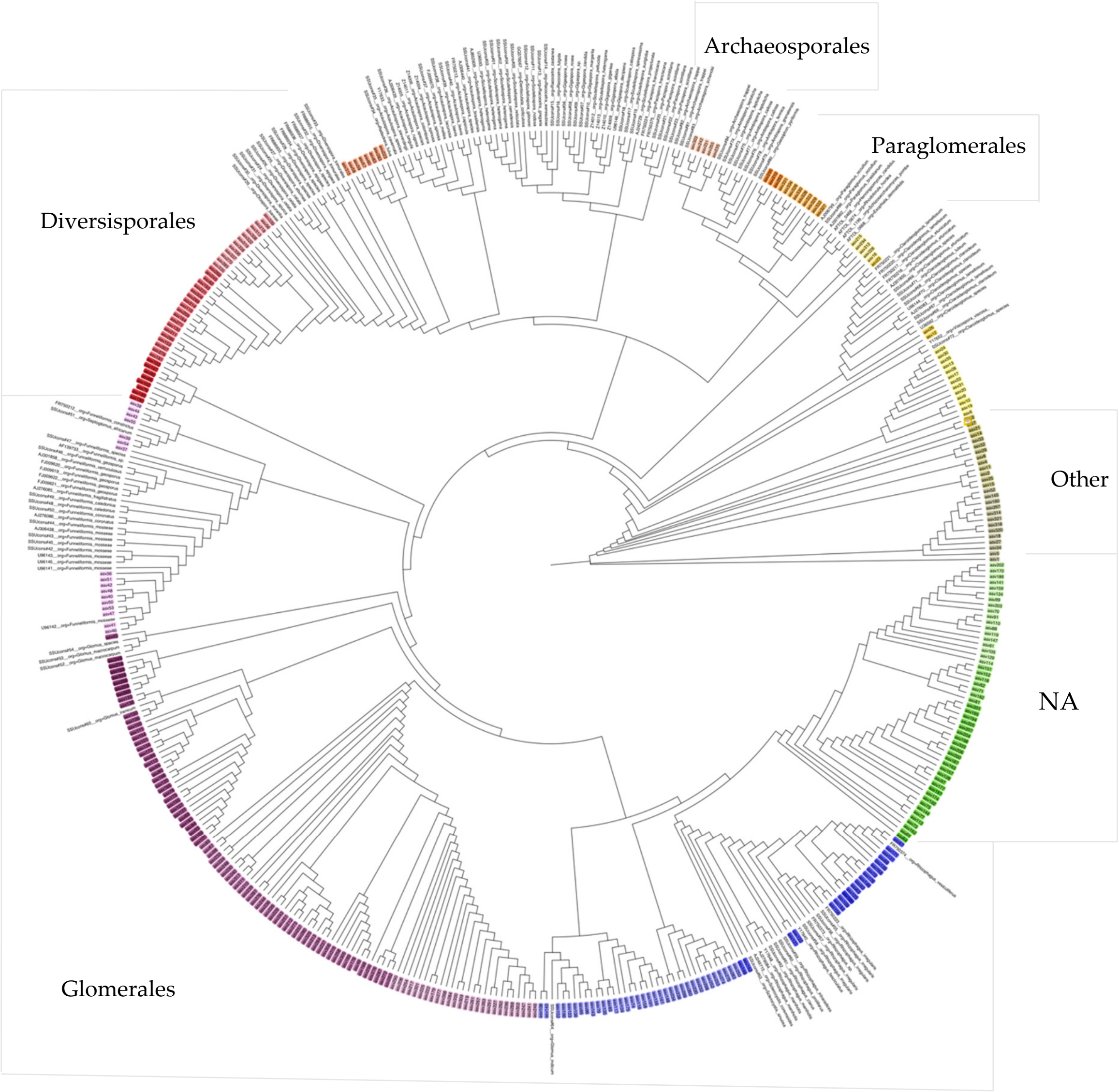

Figure S3: taxonomic assignment and RaxML phylogenetic tree of mycorrhizal AVSs Different colors are used to differentiate each family assigned. Red to pink: Diversisporales; Yellow to orange: Paraglomerales; Dark orange: Archaeosporales; Green: Non-Assigned; Blue and Purple: Glomerales and Grey: Others

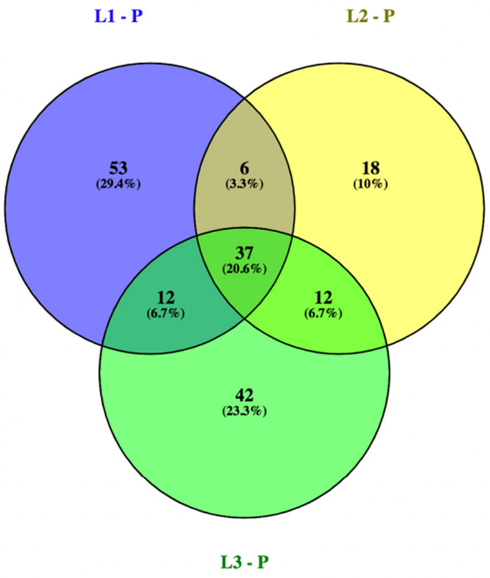

	ASV	Read
L1-P	108	80 213
L2-P	73	25 535
L3-P	103	110 219

(A)

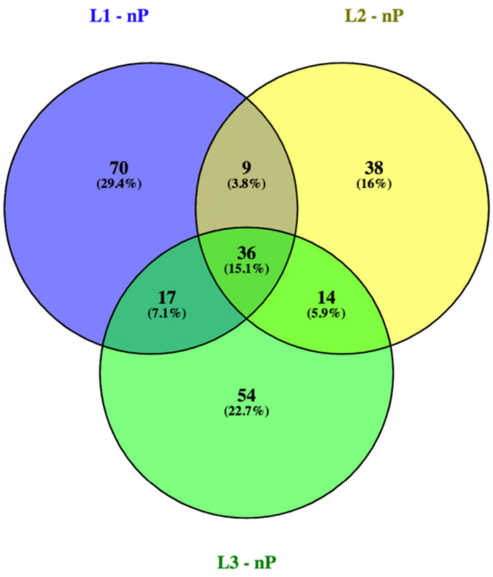

	ASV	Read
L1-nP	132	83 800
L2-nP	97	69 677
L3-nP	121	89 819

(B)

Figure S4: Numbers of specific and shared ASVs comparing (A) the P-sites and (B) the nP sites of the three localizations: Low membership characterized by a high number of ASVs specific to one site and few overlapping ASVs

Table S1: Total P and available Pi contents in soils and soil physico-chimics properties sampled across 3 localizations (L1, L2 L3), each harboring "P" sites covered in the past by PR deposits or "nP" sites without deposit

		Sites					
		**L1 - P**	**L1 - nP**	**L2 - P**	**L2 - nP**	**L3 - P**	**L3 - nP**
Available Pi (Olsen) (mg/Kg)		46.10	5.04	339.46	8.09	192.78	12.82
Total P (mg/Kg)		2860.0	1380.2	13,927.9	1067.1	10,739.1	1496.3
Ratio Pi/P %		1.61	0.36	2.43	0.75	1.79	0.85
pH(H_20)		7.058	6.956	7.552	6.868	7.324	7.267
Granulometry fractions %	Argiles	73.4	63.3	57.7	56.1	56.0	63.7
	Limons fins	14.8	20.9	15.4	22.8	16.1	13.4
	Limons grossiers	5.6	9.7	6.5	12.5	6.4	6.0
	Sables fins	3.3	3.5	6.3	4.1	8.6	9.9
	Sables grossiers	2.9	2.5	14.1	4.5	12.9	7.0
Total carbonates CT %		0.10	0.10	0.21	0.10	0.10	0.10
Organic matter by dry combustion (Dumas)	MO %	6.50	6.04	8.84	9.34	11.64	14.22
	Corg %	3.77	3.50	5.13	5.42	6.75	8.25
	N ‰	3.04	3.12	3.65	4.49	5.63	6.99
	Corg/N	12.42	11.22	14.04	12.06	12.00	11.80
Absorption complex (Co(NH3)6Cl3)	Ca me/100g	25.52	23.48	24.03	25.01	31.13	30.50
	Mg me/100g	1.05	1.14	0.72	1.26	1.15	1.55
	K me/100g	0.31	0.23	0.36	0.32	0.41	0.42
	Na me/100g	0.12	0.13	0.09	0.13	0.13	0.12
	Al me/100g	0.00	0.01	0.00	0.00	0.00	0.00
	Mn me/100g	0.01	0.02	0.01	0.03	0.01	0.01
Total element content	Fe mg/kg	50,426.5	46,905.7	66,132.3	47,679.1	50,843.3	48,395.1
	Mn mg/kg	548.9	817.3	1382.3	1407.2	1328.9	1532.8

	Al mg/kg	127,446.1	107,072.7	108,967.9	93,340.1	101,438.5	93,827.2
	Cu mg/kg	43.9	35.6	71.9	24.0	65.2	35.8
	Zn mg/kg	197.3	147.7	632.1	131.0	467.1	173.4
	Ca mg/kg	11,403.5	7896.5	45,554.7	8666.6	33942.1	14260.3
	Mg mg/kg	3482.7	3286.4	1989.5	3754.3	3244.5	3551.1
	K mg/kg	11,590.6	11,296.7	8917.8	12,588.3	12,326.4	12,271.6
	Na mg/kg	1139.9	1589.8	902.7	2039.3	2133.9	2168.8

Properties mainly measured by Inductively Coupled Plasma spectrometry-atomic emission spectrometry (ICP-AES) / OM: organic matter; $C_{org:}$ organic carbon.

In each site, analyzed soil fraction is a composite fraction obtained by pooling subsamples collected under 12 individual plants

Table S2 : Distribution of number of reads in each samples.
https://drive.google.com/drive/folders/1E8DLOBk-wnGQTGW5iCnKP3K2Z1hvoHv7?usp=sharing

Table S3 : Taxonomic distribution of the ASVs identified as AMF by the Silva database and those identified by the phylogenetics analysis.
https://drive.google.com/drive/folders/1E8DLOBk-wnGQTGW5iCnKP3K2Z1hvoHv7?usp=sharing

Table S4: Taxonomic distribution patterns of the 318 ASVs identified as Glomeromycota, at the genus (A) and species (B) taxonomic rank

A		B		
Taxonomic assignation to genus rank	ASV Numbers	Taxonomic assignation to species rank	ASV Numbers	Read Numbers
Archaeospora	5	*Archaeospora trappei*	5	3402
Claroideoglomus	3	*Claroideoglomus lamellosum*	1	18
Funneliformis	15	*Funneliformis mosseae*	10	12 591
Glomus	14	*Glomus indicum*	9	2698
Paraglomus	9	*Glomus iranicum*	4	780
Rhizophagus	18	*Rhizophagus irregularis*	2	123
Septoglomus	2	*Rhizophagus vesiculiferus*	6	8033
		Septoglomus africanum	2	270
TOTAL assigned	66 (20.7%)	TOTAL assigned	39 (12.2%)	27 915 (6%)
NA	252 (79.3%)	NA	279 (87.8%)	431 348 (94%)
TOTAL	318 ASVs	TOTAL	318 ASVs	

Table S5: ASVs with assignment up to species taxonomic level (39 ASVs)

ASV	Family	Genus	Species	Read Number
asv231	Archaeosporaceae	*Archaeospora*	*Archaeospora_trappei*	1335
asv235	Archaeosporaceae	*Archaeospora*	*Archaeospora_trappei*	1131
asv242	Archaeosporaceae	*Archaeospora*	*Archaeospora_trappei*	579
asv255	Archaeosporaceae	*Archaeospora*	*Archaeospora_trappei*	303
asv303	Archaeosporaceae	*Archaeospora*	*Archaeospora_trappei*	36
asv23	Claroideoglomeraceae	*Claroideoglomus*	*Claroideoglomus_lamellosum*	18
asv36	Glomeraceae	*Funneliformis*	*Funneliformis_mosseae*	11855
asv40	Glomeraceae	*Funneliformis*	*Funneliformis_mosseae*	164
asv41	Glomeraceae	*Funneliformis*	*Funneliformis_mosseae*	145
asv42	Glomeraceae	*Funneliformis*	*Funneliformis_mosseae*	138
asv46	Glomeraceae	*Funneliformis*	*Funneliformis_mosseae*	102
asv47	Glomeraceae	*Funneliformis*	*Funneliformis_mosseae*	81
asv48	Glomeraceae	*Funneliformis*	*Funneliformis_mosseae*	50
asv50	Glomeraceae	*Funneliformis*	*Funneliformis_mosseae*	34
asv51	Glomeraceae	*Funneliformis*	*Funneliformis_mosseae*	22
asv53	Glomeraceae	*Funneliformis*	*Funneliformis_mosseae*	19
asv106	Glomeraceae	*Glomus*	*Glomus_indicum*	384
asv108	Glomeraceae	*Glomus*	*Glomus_indicum*	348
asv123	Glomeraceae	*Glomus*	*Glomus_indicum*	277
asv132	Glomeraceae	*Glomus*	*Glomus_indicum*	227
asv140	Glomeraceae	*Glomus*	*Glomus_indicum*	142
asv143	Glomeraceae	*Glomus*	*Glomus_indicum*	132
asv196	Glomeraceae	*Glomus*	*Glomus_indicum*	9
asv95	Glomeraceae	*Glomus*	*Glomus_indicum*	596
asv96	Glomeraceae	*Glomus*	*Glomus_indicum*	583
asv126	Glomeraceae	*Glomus*	*Glomus_iranicum*	274
asv133	Glomeraceae	*Glomus*	*Glomus_iranicum*	225
asv138	Glomeraceae	*Glomus*	*Glomus_iranicum*	165
asv150	Glomeraceae	*Glomus*	*Glomus_iranicum*	116
asv156	Glomeraceae	*Rhizophagus*	*Rhizophagus_irregularis*	98
asv184	Glomeraceae	*Rhizophagus*	*Rhizophagus_irregularis*	25
asv131	Glomeraceae	*Rhizophagus*	*Rhizophagus_vesiculiferus*	228
asv139	Glomeraceae	*Rhizophagus*	*Rhizophagus_vesiculiferus*	147
asv166	Glomeraceae	*Rhizophagus*	*Rhizophagus_vesiculiferus*	52
asv171	Glomeraceae	*Rhizophagus*	*Rhizophagus_vesiculiferus*	43
asv67	Glomeraceae	*Rhizophagus*	*Rhizophagus_vesiculiferus*	3787
asv68	Glomeraceae	*Rhizophagus*	*Rhizophagus_vesiculiferus*	3776
asv39	Glomeraceae	*Septoglomus*	*Septoglomus_africanum*	258
asv54	Glomeraceae	*Septoglomus*	*Septoglomus_africanum*	12

Table S6: Shared ASVs (26) by the six sampling sites

ASVs	Family	Genus	Species	Read Number
asv61	Glomeraceae	NA	NA	78761
asv62	Glomeraceae	NA	NA	50728
asv208	Glomeraceae	NA	NA	38464
asv209	Diversisporaceae	NA	NA	17775
asv210	Glomeraceae	NA	NA	14419
asv212	Glomeraceae	NA	NA	13172
asv213	Glomeraceae	NA	NA	12331
asv214	Diversisporaceae	NA	NA	10116
asv63	Glomeraceae	*Rhizophagus*	NA	8551
asv64	Glomeraceae	NA	NA	7342
asv65	Glomeraceae	NA	NA	6481
asv217	Glomeraceae	NA	NA	6427
asv67	Glomeraceae	*Rhizophagus*	*Rhizophagus vesiculiferus*	3787
asv68	Glomeraceae	*Rhizophagus*	*Rhizophagus vesiculiferus*	3776
asv69	Glomeraceae	NA	NA	3288
asv222	Glomeraceae	NA	NA	3279
asv70	Glomeraceae	NA	NA	2955
asv74	Glomeraceae	NA	NA	2027
asv226	Glomeraceae	NA	NA	1992
asv77	Glomeraceae	NA	NA	1821
asv229	Glomeraceae	NA	NA	1763
asv230	Glomeraceae	NA	NA	1524
asv237	Glomeraceae	NA	NA	1119
asv240	Glomeraceae	NA	NA	720
asv92	Glomeraceae	*Rhizophagus*	NA	652
asv97	Glomeraceae	NA	NA	575

Table S7: ASVs identified after indicator species analysis

ASV	Group	p_values	Order	Family	Species	Read Total	ASV also referred to as:
P or nP group analysis							
asv131	nP	0.0492*	Glomerales	Glomeraceae	*R. vesiculiferus*	228	nP Specific ASV
asv208	nP	0.0194*	Glomerales	Glomeraceae	NA	38,464	Microbial core ASV
asv219	nP	0.0472*	Glomerales	Glomeraceae	NA	3988	nP Specific ASV
asv248	P	0.0081**	Glomerales	Glomeraceae	NA	384	Shared ASV
asv69	nP	0.0113*	Glomerales	Glomeraceae	NA	3288	Microbial core ASV
asv77	nP	0.0111*	Glomerales	Glomeraceae	NA	1821	Microbial core ASV
asv80	nP	0.0053**	Glomerales	Glomeraceae	NA	1593	Shared ASV
asv84	nP	0.0237*	Glomerales	Glomeraceae	NA	1040	nP Specific ASV
P, nP, S or R group analysis							
asv101	RnP	0.0496*	Glomerales	Glomeraceae	NA	491	nP Specific ASV
asv134	RnP	0.0110*	Glomerales	Glomeraceae	NA	203	nP Specific ASV
asv236	RnP	0.0485*	Glomerales	Glomeraceae	NA	1122	Shared ASV
asv264	RP	0.014*	Glomerales	Glomeraceae	NA	212	Shared ASV
asv69	RnP	0.0024**	Glomerales	Glomeraceae	NA	3288	Microbial core ASV
asv78	RnP	0.0017**	Glomerales	Glomeraceae	NA	1802	Shared ASV
asv80	RnP	0.0218*	Glomerales	Glomeraceae	NA	1593	Shared ASV
asv84	RnP	0.0271*	Glomerales	Glomeraceae	NA	1040	nP Specific ASV
asv93	RnP	0.0041**	Glomerales	Glomeraceae	NA	650	Shared ASV
asv116	SnP	0.0494*	Glomerales	Glomeraceae	NA	309	Shared ASV
asv208	SnP	0.0413*	Glomerales	Glomeraceae	NA	38,464	Microbial core ASV
asv216	SnP	0.0139*	Glomerales	Glomeraceae	NA	6440	Shared ASV
asv235	SnP	0.0093**	Archaeosporales	Archaeosporaceae	*A. trappei*	1131	nP Specific ASV
asv255	SnP	0.0454*	Archaeosporales	Archaeosporaceae	*A. trappei*	303	nP Specific ASV
asv258	SnP	0.0055**	Glomerales	Glomeraceae	NA	267	Shared ASV
asv66	SnP	0.0292*	Glomerales	Glomeraceae	NA	4400	Shared ASV
asv76	SnP	0.0113	Glomerales	Glomeraceae	NA	1868	Shared ASV
asv77	SnP	0.0045**	Glomerales	Glomeraceae	NA	1821	Microbial core ASV
asv214	SP	0.0280*	Diversisporales	Diversisporaceae	NA	10,116	Microbial core ASV
asv88	SP	0.0420*	Glomerales	Glomeraceae	NA	741	Shared ASV
asv91	SP	0.0186*	Glomerales	Glomeraceae	NA	653	Shared ASV
asv97	SP	0.0095**	Glomerales	Glomeraceae	NA	575	Microbial core ASV
asv222	RnP+RP	0.0019**	Glomerales	Glomeraceae	NA	3279	Shared ASV
asv237	RnP+RP	0.0157*	Glomerales	Glomeraceae	NA	1119	Shared ASV
asv97	RnP+RP	0.0296*	Glomerales	Glomeraceae	NA	575	Microbial core ASV
asv327	SnP+SP	0.0069**	Paraglomerales	Paraglomeraceae	NA	2200	Shared ASV
asv61	SnP+SP	0.0346	Glomerales	Glomeraceae	NA	78,761	Microbial core ASV
asv62	SnP+SP	0.0236	Glomerales	Glomeraceae	NA	50,728	Microbial core ASV
